# MiR-1246b, a novel miRNA molecule of extracellular vesicles in bronchoalveolar lavage fluid, promotes nodule growth through FGF14 in patients with lung cancer

**DOI:** 10.1038/s41419-023-06218-9

**Published:** 2023-12-01

**Authors:** Jing Huang, Ming Ding, Yuan Lu, Lu Xu, Yu Zhang, Shuhua Han, Xiaoli Zhu, Yiping Li, Pingsheng Chen

**Affiliations:** 1 Department of Respiratory and Critical Care Medicine, Zhongda Hospital, School of Medicine, Southeast University, Nanjing, Jiangsu China; 2 Department of Pathology, School of Medicine, Southeast University, Nanjing, Jiangsu China

**Keywords:** Lung cancer, Diagnostic markers

## Abstract

With the widespread development of chest computed tomography (CT), the detection rate of pulmonary nodules has increased; therefore, the classification of benign vs. malignant nodules has become a common problem in the clinic. MicroRNA, a potential tool, is expected to become a good choice for diagnosing and studying the occurrence and development of diseases through the vector of bronchoalveolar lavage fluid extracellular vesicles (BALF-EVs). In this study, radial endobronchial ultrasound (R-EBUS) was used to locate pulmonary nodules in patients. BALF was obtained, EVs were isolated, and small RNA sequencing was performed to screen differentially expressed miRNAs between benign and malignant pulmonary nodules. The binding targets and underlying mechanisms of the differentially expressed miRNAs were verified by in vitro and in vivo experiments. R-EBUS localization and sampling was used to obtain BALF, and EVs were successfully isolated and characterized. Differentially expressed miRNAs in BALF-EVs of patients with benign vs. malignant pulmonary nodules were screened by high-throughput small RNA sequencing. A new miRNA, miR-1246b, was identified. We found that FGF14 was the binding target of miR-1246b by luciferase assay. Subsequent mechanistic studies showed that miR-1246b inhibited the expression of FGF14 in lung cancer cells, further leading to ERK phosphorylation and epithelial-to-mesenchymal transition (EMT), which ultimately contributed to lung cancer cell proliferation, migration and invasion. In summary, our study demonstrates that the detection of miRNAs in BALF-EVs, a means of liquid biopsy, could assist in distinguishing malignant nodules from benign nodules. miR-1246b, which was extracted from BALF-EVs, targets FGF14 to promote lung cancer cell proliferation, migration and invasion.

## Introduction

Due to the high morbidity and mortality of lung cancer, chest low-dose computed tomography (LDCT) is recommended for screening in international clinical guidelines [1]. However, with the widespread use of LDCT, the detection rate of pulmonary nodules has been increasing significantly. Due to the small sizes of pulmonary nodules and the main distribution in the periphery of the lung, it is very difficult to make a definitive diagnosis. For pulmonary nodules without surgical indications, respiratory interventional techniques such as percutaneous lung puncture or transbronchial lung biopsy are often used to assist in the diagnosis, but even with the combined use of multiple techniques, the overall diagnostic coincidence rate for isolated peripheral pulmonary lesions is 62–92% [2, 3]. There are still some patients who cannot be diagnosed, and the medical cost has increased significantly. Therefore, liquid biopsy has become a research hotspot in the diagnosis of pulmonary nodules. Technology for detecting circulating tumor cells and circulating tumor DNA has developed rapidly and has been used for the detection of lung cancer, the evaluation of treatment effects and the prediction of prognosis in patients with lung cancer in clinical practice [4, 5]. However, peripheral blood samples are used to detect both these markers, and blood components can significantly affect the results. The specificity in the diagnosis of early lung cancer is poor [6, 7], and sequencing has high economic costs.

As a main carrier and transmitter of intercellular signals, extracellular vesicles (EVs) contain a variety of biological information on donor cells and may be more specific for tumor diagnosis [8]. MicroRNAs (miRNAs) have been found to be enriched to varying degrees in EVs and can be used as a potential tool to diagnose lung cancer, determine the prognosis of patients, and monitor treatment effects [9, 10]. Previous studies have shown that EVs isolated from the blood of lung cancer patients contain miR-210 and miR-23a, which promote tumor angiogenesis [11, 12]. miR-23a also regulates transforming growth factor-β (TGF-β)-mediated epithelial-to-mesenchymal transition (EMT) by targeting cadherin [13]. However, the research samples from these studies were also derived from blood, and there was poor specificity. Furthermore, most studies only include patients with advanced tumors, and few studies are for early-stage lung cancer.

Bronchoalveolar lavage fluid (BALF) can directly contact lung lesions and thus contains various components of peripheral lung tissue (for example, phospholipids and soluble peptides such as proteins and nucleic acids) and small airway cells [14]. Some studies have proposed that BALF can be used for tumor DNA mutation/methylation detection as a biomarker for lung cancer diagnosis, but the positive detection rate for adenocarcinoma is only 69.6% [15], and lung cancer presenting peripheral nodules is mostly adenocarcinoma. There are many studies on the mechanisms related to BALF-EVs in acute and chronic airway inflammatory diseases, including asthma, acute respiratory distress syndrome (ARDS), chronic obstructive pulmonary disease, pulmonary sarcoidosis and pulmonary fibrosis [16–19]. However, studies on lung cancer are rare. Mass spectrometry-based proteomics in BALF-EVs from lung cancer patients has revealed high proteomic complexity [20]. BALF-EVs have also been used to detect driver genes such as EGFR and biomarkers of drug resistance to guide treatment instead of tissue biopsy [21, 22], but the individuals included in these studies were mostly advanced lung cancer patients. Some previous studies also reported abnormal miR-107, miR-126, miR-124-3p, miR-7-5p, miR-424-5p, miR-196b-5p, miR-18-5p, miR-196b-5p, miR-185–5p, miR-18a-5p and Let-7a expression in lung cancer patients [23–25]; however, only the Kim group focused on early-stage lung cancer [25].

In this study, guided by radial endobronchial ultrasound (R-EBUS), local BALF was obtained from patients with pulmonary nodules, and EVs were isolated. Using high-throughput sequencing, a new miRNA with a large expression difference in BALF-EVs between benign and malignant nodules, miR-1246b, was identified. The underlying molecular mechanisms were initially explored through the validation of its binding targets in lung cancer.

## Subjects and methods

### Patient data collection

Patients who underwent bronchoscopy for pulmonary nodules at Zhongda Hospital, Southeast University, from August 2021 to January 2022 were eligible for inclusion. The inclusion criteria were as follows: age of more than 18 years; pulmonary nodules found on computed tomography (CT); signed informed consent form; willingness to cooperate with the study; and complete and comprehensive clinicopathological data. The exclusion criteria were as follows: active pulmonary tuberculosis; grade IV cardiac function and/or acute myocardial infarction within 3 months; unhealed hematological diseases or malignant tumors; pregnant and lactating women; forced expiratory volume in the first second (FEV1) < 35% due to decreased pulmonary function; acceptance of tumor therapy such as surgery, chemotherapy, radiotherapy, and immunotherapy; and inability to cooperate or refusal of bronchoscopy. Each patient had pathological sections prepared after bronchoscopy biopsy or surgical resection. The diagnosis of malignant vs. benign pulmonary nodules was performed via pathological examination. Infection was considered a causative factor in cases in which the pathogen was clearly detected and/or the nodules disappeared after antibiotic treatment. The study necessitated the random selection of 50 patients who met the inclusion and exclusion criteria from the diagnosed patient pool, comprising equal proportions of benign and malignant cases (25 each). This study, which involved human participants, was reviewed and approved by the Ethics Committee of Zhongda Hospital, Southeast University (ethics approval number 2021ZDSYLL156-P).

### BALF acquisition

After the patient underwent monitored anesthesia care (MAC), R-EBUS was employed to locate the lesion and guide BALF collection. Following the “Chinese Expert Consensus Statement on Issues Related to Small Specimen Sampling of Lung Cancer” [26], a 60 ml syringe was used to perfuse 0.9% sterile sodium chloride solution at room temperature, which was injected through a bronchoscope into the affected lung tissue; BALF was aspirated into a sterile container via bronchoscopy and kept at 4 °C.

### EV isolation, purification and identification

To isolate EVs using ultracentrifugation, BALF was first transferred to a centrifuge tube and centrifuged at 4 °C at 300 × *g* for 5 min. Then, the pellet was examined cytologically, and the supernatant was centrifuged at 2000 × *g* for 10 min. Next, the supernatant was centrifuged at 12,000 × *g* for 30 min and 120,000 × *g* for 2 h. Finally, the EV precipitate was resuspended in sterile PBS for further use.

To identify EVs, 10 μl of resuspended EVs was added to 10 μl of 4% paraformaldehyde, and the mix was added onto a carbon film (200 mesh, Beijing Zhongjingkeyi Technology Co., Ltd., China). Following standing for 5 min, the mix was incubated in uranyl negative staining solution for 10 min after draining on filter paper and observed by transmission electron microscopy (Hitachi HT7700, Japan). Twenty microliters of EVs were dissolved in 1 ml of PBS, and the solution was vortexed for 1 min to keep EVs evenly distributed. EV particle size distribution (PSD) was then assessed using a particle size analyzer (ZetaView PMX‐120, Particle Metrix, Germany) at ×10 magnification with software version 8.05.12. The instrument settings were 16 °C, gain of 19 and shutter of 100. Measurements were performed at 11 different positions (two cycles per position) and a frame rate of 30 frames per second. Image evaluation was performed on particles with minimum brightness: 20, minimum area: 5, maximum area: 1000, and maximum brightness: 255. The recognition radius was 100, and the minimum track length was 15. The protein markers of EVs, i.e., CD63, ALIX and calnexin, were identified by western blotting.

### Cell culture

The lung adenocarcinoma cell lines H1299, H1975 and A549 and the HEK293T cell line were purchased from Key GEN BioTECH. HK-2 cells were generously provided by the Renal Disease Research Institute, Zhongda Hospital, Southeast University. All cells were cultured in incomplete RPMI-1640 medium (with double antibiotics)(KGM31800-500, Key GEN BioTECH, China) containing 10% fetal bovine serum (Every Green, Zhejiang Tianhang Biotechnology CO. LTD, China) and 1% streptomycin-penicillin (Beyotime, China). The cells were cultured in an incubator at 37 °C with 5% carbon dioxide. All cell lines were authenticated by STR profiling and routinely examined to be mycoplasma-free using the mycoplasma detection kit (KGY011, Key GEN BioTECH, China).

### Tracer experiment

BALF-EVs were resuspended in 1 ml diluent C, and 6 μl PKH67 was added; the sample was mixed and incubated at 37 °C for 5 min, and centrifuged at 120,000 × *g* for 2 h. The precipitate was resuspended in PBS for washing. Approximately 5 × 10^5^ cells were seeded in each well of 6-well plate, 200 μl labeled BALF-EVs were added to the cells, and the cells were cultured for 24 h; then, the cells were washed with PBS 3 times. Then, 100 µl of prepared Hoechst staining solution was added to each well, and the cells were placed at room temperature and kept out of light for 30 min; EVs uptake in cells was observed under laser confocal microscope (600×); blue represents the nucleus, and green represents EVs.

### Cell transfection

miR-1246b mimics, inhibitors, negative controls and plasmids were synthesized by GenePharma (Shanghai, China) (mimic – forward: 5′-AUGGAUUUUUGGAGCAGGG-3′, reverse: 5′-CCCUGCUCCAAAAAUCCAU-3′; inhibitor – 5′- CCCUGCUCCAAAAAUCCAU-3′. Cells were seeded in 6-well plates and transfected using Lipofectamine 3000 reagent (Invitrogen Thermo Fisher Scientific, Inc. USA) following the manufacturer’s instructions. Transfected cells were cultured for 48 h and harvested for later use.

### Quantitative real-time PCR

Total RNA from EVs or cells was extracted using TRIzol (15596-026, Invitrogen, USA). mRNA was reverse transcribed with prime script RT Master Mix, and miRNA expression was detected by a TB Green™ Premix Ex Taq™ II kit (TaKaRa RR820B, Japan). miRNA levels were normalized to U6, and relative expression was calculated using the 2^−ΔΔCt^ method. Primers were provided by GENERAL Biosystems (Anhui) Co., Ltd. and are listed in Table 1.

**Table 1:** Primer sequences.

Name	Primer sequences
miR-1246b	Forward: 5′-GCGCGATGGATTTTTGGA-3′
	Reverse: 5′-AGTGCAGGGTCCGAGGTATT-3′
FGF14	Forward: 5′-TGGAACCAAGGATGACAGCACT-3′
	Reverse: 5′-TCACTCCCTGGATGGCAACAAC-3′
ERK	Forward: 5′-CGGCATGGTGTGCTCTGCTTAT-3′
	Reverse: 5′-TGGCAGTAGGTCTGGTGCTCAA-3′
E-cadherin	Forward: 5′-CTACAATGCCGCCATCGCTTAC-3′
	Reverse: 5′-GGTGACCACACTGATGACTCCT-3′
N-cadherin	Forward: 5′-CCTGAGGGATCAAAGCCTGGAA-3′
	Reverse: 5′-TGGAGCCTGAGACACGATTCTG-3′
Vimentin	Forward: 5′-TGCAGGACTCGGTGGACTTCTC-3′
	Reverse: 5′-AGTTGGCGAAGCGGTCATTCAG-3′
U6	Forward: 5′-CTCGCTTCGGCAGCACA-3′
	Reverse: 5′-AACGCTTCACGAATTTGCGT-3′
GAPDH	Forward: 5′-AGATCATCAGCAATGCCTCCT-3′
	Reverse: 5′-TGAGTCCTTCCACGATACCAA-3′

### Dual-luciferase reporter gene assay

Bioinformatics analysis based on the miRanda and PITA databases revealed that miR-1246b has a potential binding site in the 3ʹ-UTR of FGF14. The pmirGLO vector was used to demonstrate direct binding between miR-1246b and the 3ʹ-UTR of FGF14. pmirGLO-miR-1246b mimic, pmirGLO-FGF14-WT+miR-1246b mimic, pmirGLO FGF14-MUT+miR-1246b mimic, pmirGLO-miR-1246b inhibitor, pmirGLO-FGF14-WT+miR-1246b inhibitor, pmirGLO-FGF14-MUT+miR-1246b inhibitor reporter genes and a negative control (NC) were constructed to transfect HEK293T cells. After transfection for 24 h, fluorescence was measured using the Dual-Glo® luciferase detection system (Promega E2920, USA) following the manufacturer’s instructions. Relative luciferase activity was calculated as the ratio of firefly luciferase activity to Renilla luciferase activity.

### Western blot analysis

Total protein was isolated with a protein extraction kit (KGP250, Key GEN BioTECH, China), and then, the protein concentration was examined using the bicinchoninic acid assay (BCA) method. Samples (20 μg of protein in each sample) were separated by SDS-PAGE. After proteins were transferred onto PVDF membranes, the membranes were blocked in 5% skim milk for 1 h at room temperature and incubated with primary antibody overnight at 4 °C. After washing with TBST, the membranes were incubated with secondary antibody for 1 h at room temperature. Finally, ECL reagent (KGP116, Key GEN BioTECH, China) was used for development. The grayscale values of the protein bands were determined with Gel-Pro32 software (Media Cybernetics, USA), and GAPDH was used as a control. The following antibodies were used in our study: anti-CD63 monoclonal antibody (67605-1-Ig, Proteintech, Wuhan Sanying, China), anti-calnexin polyclonal antibody (10427-2-AP, Proteintech, Wuhan Sanying, China), anti-ALIX polyclonal antibody (12422-1-AP, Proteintech, Wuhan Sanying, China), anti-FGF14 antibody (bs-9761R, Bioss, China), anti-E-cadherin antibody (ab40772, Abcam, UK), anti-N-cadherin antibody (ab98952, Abcam, UK), anti-vimentin antibody (ab92547, Abcam, UK), anti-ERK antibody (ab17942, Abcam, UK), and anti-p-ERK antibody (ab201015,Abcam, UK).

### Cell proliferation

EdU test: Transfected cells were seeded in a 96-well plate at a density of 2 × 10^3^ cells/well, and EdU was added following the instructions of the keyFluor 594 Click-iT EdU Imaging Detection Kit (KGA333, Key GEN BioTECH, China). After incubation for 2 h, the cells were washed with PBS, fixed with 4% paraformaldehyde, and incubated with glycine and 1×Apollo® staining reaction solution in the dark. Next, the cells were permeabilized with 0.5% Triton X-100 in PBS, incubated in 1× Hoechst 33342 reaction solution in the dark for 30 min, and then visualized using a cell imaging system.

CCK-8 assay: Transfected cells were seeded in a 96-well plate at a density of 2 × 10^3^ cells/well and incubated for 24 or 48 h. Cell Counting Kit-8 solution (KGA317, Key GEN BioTECH, China) was added and incubated for 4 h, and the absorbance was measured at 450 nm.

### Wound healing assay

Cells were seeded into 6-well plates and grown to 90% confluence in medium containing 10% FBS. A horizontal line was drawn across the cells with a 200-μl micropipette tip. After washing away cell debris with PBS, the cells were cultured for 24 h in fresh medium. Images were taken at 0 h and 24 h. Wound healing rate = [(wound area at 0 h − wound area at 24 h)/wound area at 0 h] × 100%.

### Transwell invasion assay

Matrigel (Becton, Dickinson and Company) was diluted (1:10) in serum-free DMEM. Diluted Matrigel (100 μl) was added to the upper well of a Transwell chamber, and the chamber stood overnight. The cells were then seeded at 5 × 10^4^ into the Matrigel-coated upper chamber, and 600 μl of DMEM containing 10% FBS was added to the lower chamber. After 24 h of incubation, the cells were fixed with 4% paraformaldehyde, stained with 1% crystal violet solution for 10 min, observed and photographed using an inverted optical microscope (Nikon ECLIPSE TE2000-U, Japan). Positive cells were counted.

### Immunohistochemical staining

The tissue to be examined was fixed in formalin, embedded in paraffin and sectioned. Sections were cleared in xylene and dehydrated in a graded ethanol series, followed by antigen retrieval. Following the instructions provided by the MaxVision kit (KIT-5010, Maxin Biotech, LTD., China), 3% H_2_O_2_-methanol solution was added dropwise to the sections, and then, the sections were blocked at room temperature for 10 min. Then, 50–100 µL of 1% BSA was added dropwise to the sections, followed by incubation at room temperature for 20 min. Then, 100 μl of primary antibody was introduced dropwise to the sections, and the sections were incubated overnight at 4 °C. Goat anti-rabbit/mouse polymer (50 µl) was dropped onto the sections, which were incubated in a wet box at room temperature for 20 min. DAB solution was used for visualization, and the sections were counterstained with hematoxylin. Images were scanned with a digital pathology slide scanner (Olympus VS200, Japan) and analyzed using Image-Pro Plus 6.0 (Media Cybernetics, USA).

### Xenograft model establishment

Four-week-old NSG mice (female) were purchased from Shanghai Model Organisms Center, Inc. Cultured H1299 human lung cancer cells (0.1 ml; 2 × 10^7^ cells/ml) were subcutaneously injected into the right axilla of each mouse to establish a lung cancer xenograft model. Mice were randomly divided into five groups with five mice per group. Then, miR-1246b agomir and its NC or miR-1246b antagomir and its NC were injected into xenograft tumors (*n* = 5) twice a week (2.5 nmol dose), and tumor volume was recorded every 2 days for 3 consecutive weeks. The mice were sacrificed, the entire tumor was completely excised and weighed, and the size was measured. No blinding method was used for animal experiments. There were no animal exclusion criteria. All animal experiments were reviewed by Nanjing Ramda Pharmaceutical Co., Ltd. to ensure compliance with experimental animal ethics and were completed in accordance with institutional rules.

### Statistical analysis

SPSS 25.0 (IBM, USA) was used for the statistical analyses. Enumeration data were analyzed using the chi-square test and Spearman’s rank correlation test. All continuous variables were tested for normal distribution. Unpaired Student’s *t* tests were performed for comparisons of two groups. For comparisons of multiple groups, one-way ANOVA corrected for multiple comparisons using Dunnett’s or Tukey’s tests was performed. All results are the mean of at least three independent experiments, and data are expressed as the mean ± standard deviation. A receiver operating characteristic curve (ROC curve) was drawn for miRNA detection results. *P* value < 0.05 was considered to indicate a statistically significant difference.

## Results

### Collection and identification of BALF-EVs in patients with pulmonary nodules

**Fig. 1:**
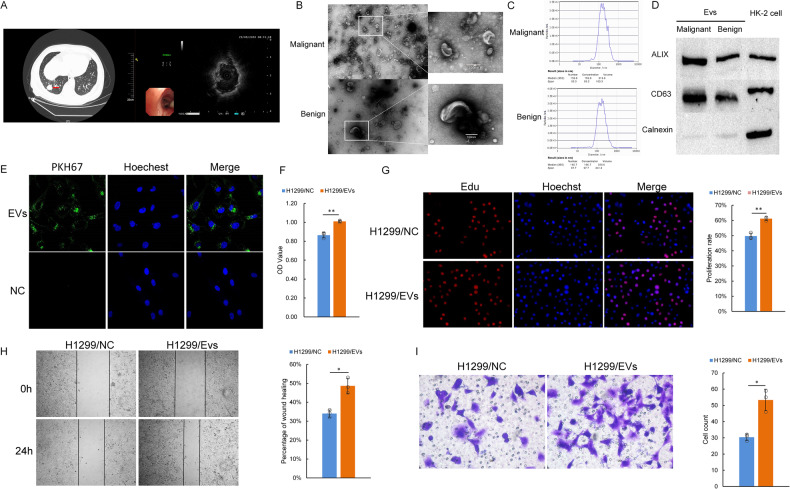
The collection, characterization and effect of BALF-EVs on the proliferation and invasion of lung cancer cells. **A** Chest CT showing a right lower pulmonary nodule (~2.5 cm), as indicated by the red arrow. The R-EBUS image of the right lower pulmonary nodule shows an abnormal echo area, indicating that the ultrasound probe has reached the lesion. **B** Morphology of BALF-EVs in BALF samples under an electron microscope (80 kV, ×10.0 K), Bar = 100 nm. **C** ZetaView measurement of EV PSD in BALF samples. **D** western blot detection of EV markers. **E** Malignant BALF-EV uptake by H1299 cells was evaluated under a laser confocal microscope (600×) in H1299 cells. Blue indicates the nucleus, and green indicates EVs. **F**, **G** EV effect on proliferation according to CCK-8 (**F**) and EdU assays (**G**) in H1299 cells. **H** A wound healing assay was performed on H1299 cells treated as indicated. **I** Transwell assays were performed on H1299 cells treated as indicated. In (**F**–**I**), a group without EVs, including culture medium only, was used as a negative control. In (**B**–**I**), the results are representative of three independent experiments. The data are presented as the means ± SDs. *P < 0.05, **P < 0.01 (Student’s *t* test).

A total of 50 patients (25 with benign nodules and 25 with malignant nodules) were enrolled in this study. The patients ranged in age from 36 to 85 years and included 22 males and 28 females. R-EBUS was performed in each patient, and BALF was obtained after probing the abnormal pulmonary echo area (Fig. 1A). The 25 malignant nodules included 23 cases of adenocarcinoma, 1 case of squamous cell carcinoma, and 1 case of small cell carcinoma. Six samples (3 for malignant and 3 for benign) were used for high-throughput sequencing, and 4 (2 for malignant and 2 for benign) were used for EV characterization. The general information of the patients, nodule sizes, pathology, and tumor staging of the remaining 40 samples are shown in Table 2.

**Table 2:** Characteristics of the patients.

Item	*n*
Sex	*n* = 40
Male (%)	19 (38%)
Female (%)	21 (42%)
Age (years, mean ± SD, range)	65.52 ± 12.51, (36–85)
Malignant	*n* = 20
Histological type	
Adenocarcinoma	18
Small cell carcinoma	1
Squamous cell carcinoma	1
TNM	
I	18
II	2
Benign	*n* = 20
Sarcoidosis	1
Inflammation	17
Aspergillosis	1
Mycobacteriosis avium	1

*SD* standard deviation.

EVs were isolated from BALF by ultracentrifugation. To characterize EVs, transmission electron microscopy (TEM) was used to visualize the bilayer structure of EVs (Fig. 1B). The size of EVs was measured by ZetaView analysis, with an average size of ~160 nm and a concentration of 1.1 × 10^8^ particles/ml (Fig. 1C). Western blot analysis revealed the presence of the marker proteins CD63 and ALIX in EVs from both groups. However, calnexin, a typical negative marker, was not detected in EVs (Fig. 1D). The above results indicated that EVs were successfully isolated from BALF. We have submitted all relevant data of our experiments to the EV-TRACK knowledgebase (EV-TRACK ID: EV230977).

The obtained EVs (extracted protein concentration 20 μg/μl) were cocultured with H1299 cells for 24 h to observe EV uptake by tumor cells (Fig. 1E). Malignant BALF-EVs were cocultured with H1299 cells, and the control group was treated with an equal volume of culture medium without EVs. The results of CCK-8, EdU, wound healing and Transwell assays showed that the proliferation, migration and invasion of H1299 cells were significantly enhanced after EV uptake (Fig. 1F–I).

### miR-1246b is upregulated in BALF-EVs from patients with malignant pulmonary nodules, and FGF14 is a direct target of miR-1246b

**Fig. 2:**
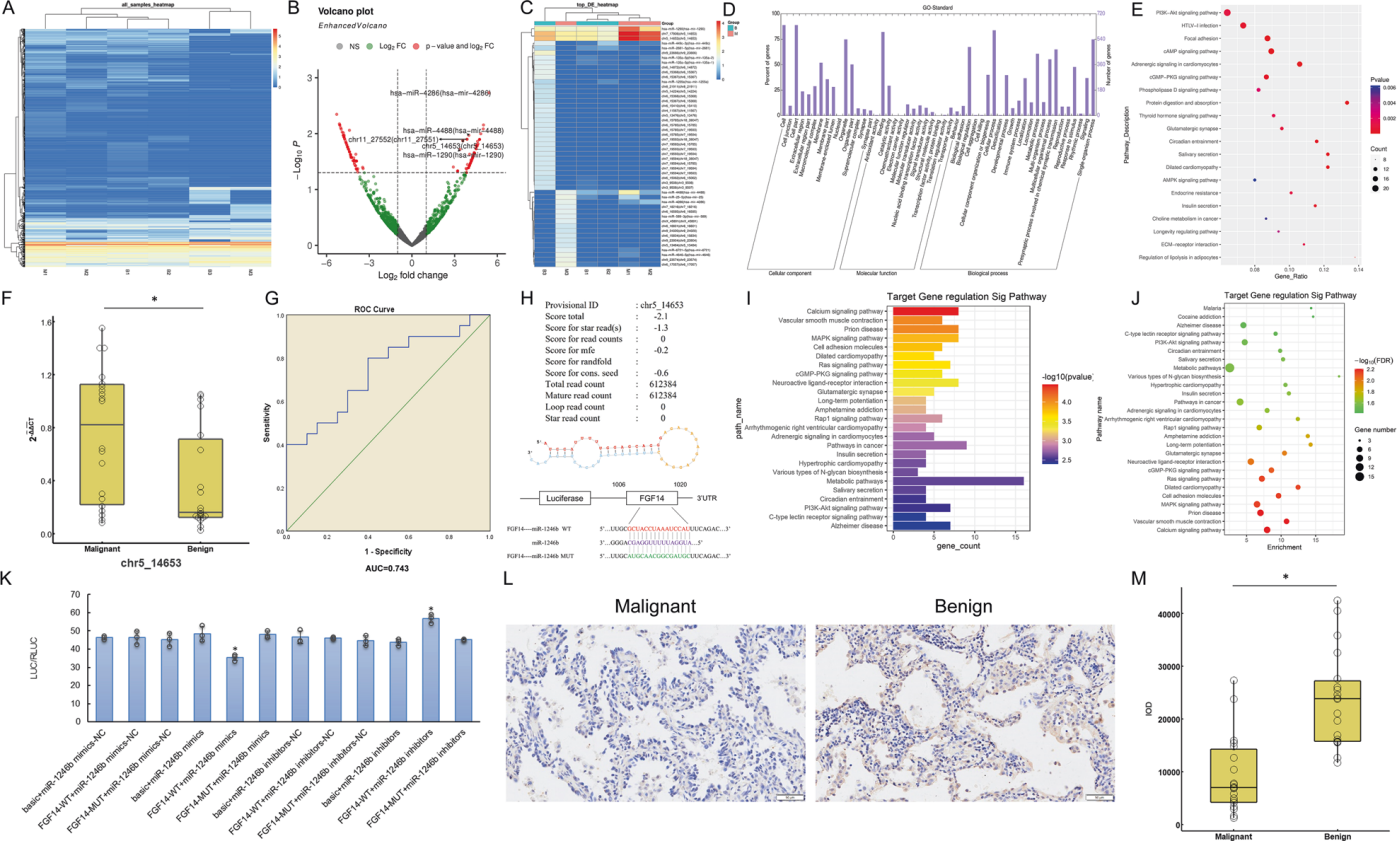
High-throughput sequencing and verification of small RNAs in BALF-EVs. **A** Cluster analysis of EV samples. The log10 values of miRNAs in the samples were used for cluster analysis. **B** The differential gene volcano plot showing the overall differential gene distribution. The threshold was set to |logFC | >1 and FDR < 0.05. The genes of interest are marked with arrows and accompanied by their locus labels/gene names. **C** Hierarchical clustering diagram of differentially expressed miRNAs. The vertical column represents the sample, each row represents a miRNA, the right side is marked with the miRNA name, and the expression level gradually decreases from red to blue. **D** GO enrichment differences for the target genes of newly identified miRNAs. **E** KEGG enrichment analysis of the target genes of newly identified miRNAs. The top 10 positions were selected for pathway enrichment analysis. **F** Chr5_14653 expression assessed by qPCR in BALF-EV samples. The results showed that the level of chr5_14653 was higher in malignant nodules than in benign nodules. **G** ROC analysis indicated that the AUC for chr5_14653 in the diagnosis of benign and malignant nodules was 0.743. **H** Schematic diagram of the chr5_14653 (miR-1246b) structure and its FGF14 binding site. **I**, **J** Histograms and dot plots of signaling pathways predicted by the miRanda and PITA databases. **K** The dual-luciferase assay results indicated that the LUC/RLUC values for FGF14-WT+miRNA mimic were significantly lower than those for the other groups, suggesting that FGF-14 is the target gene of miR-1246b. **L**, **M** FGF14 expression examined by immunohistochemistry. The left picture shows a typical pathological image from stage IA adenocarcinoma, and the right picture is from an inflammatory nodule. M shows the quantitative analysis of the immunohistochemistry results. In (**K**), the results are representative of three independent experiments (One-way ANOVA); In (**F** and **M**), number of samples = 20 (Student’s *t* test). The data are presented as the means ± SDs. *P < 0.05.

To determine miRNA expression differences in BALF-EVs from patients with benign or malignant pulmonary nodules, we randomly selected 3 benign and 3 malignant pulmonary nodules for high-throughput sequencing of small RNAs. Differentially expressed miRNAs were present in BALF-EVs from patients with benign and malignant samples (Fig. 2A–E). On the basis of the cluster analysis results, 5 miRNAs that were upregulated in malignant nodules were selected, and qRT‒PCR of 20 pairs of BALF-EV samples from patients with benign and malignant nodules was used for validation. The expression level of chr5_14653, a novel miRNA, was found to be higher in patients with malignant nodules than in patients with benign nodules. In the ROC analysis, the AUC was 0.743, and the sensitivity and specificity values were 80% and 60%, respectively (Fig. 2F, G). According to mRNA naming rules, chr5_14653 was named miR-1246b.

Analyses using the miRanda and PITA databases predicted FGF14 as a target of miR-1246b (Fig. 2H–J). To confirm whether miR-1246b directly regulates FGF14, dual-luciferase reporter experiments were conducted. In HEK293T cells, miR-1246b expression significantly inhibited the luciferase activity of the wild-type (WT) FGF14 3′UTR but not that of the mutant (MUT) FGF14 3′UTR; in contrast, inhibiting miR-1246b significantly increased the luciferase activity of the WT-FGF14 3′UTR, but the luciferase activity of the MUT-FGF14 3′UTR was unchanged (Fig. 2K), confirming the existence of a binding relationship between miR-1246b and FGF14. The expression of FGF14 protein was assessed in 20 pairs of benign and malignant lung nodule samples, and the results showed that FGF14 was highly expressed in malignant lung nodule tissue (Fig. 2L, M).

### miR-1246b promotes the proliferation, migration and invasion of lung cancer cells

**Fig. 3:**
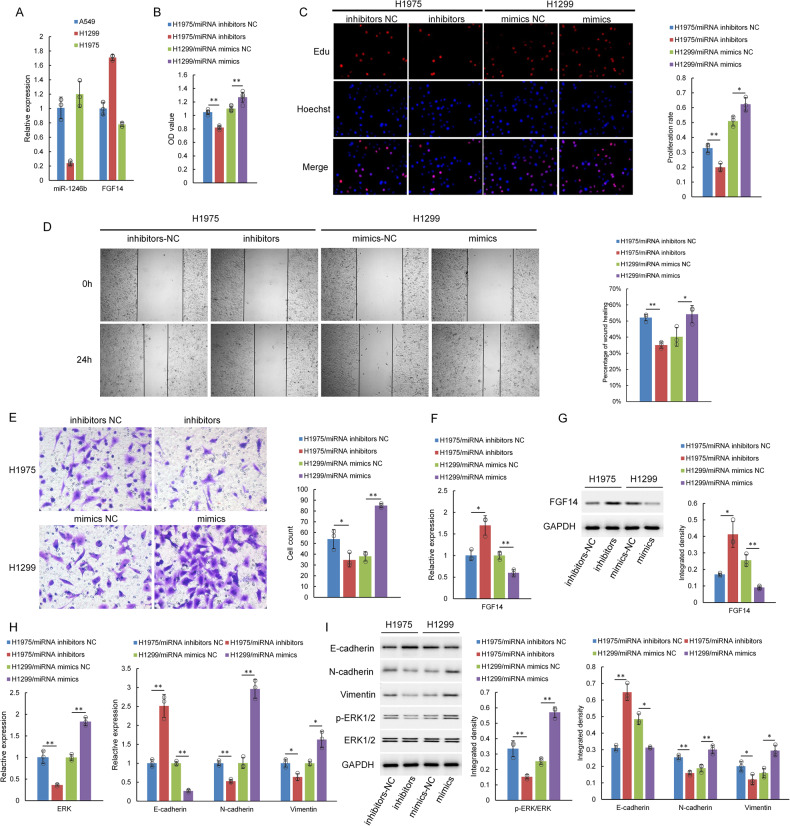
miR-1246b may promote cell proliferation, migration and invasion by inhibiting FGF14. **A** miR-1246b and FGF14 mRNA levels were determined by qPCR in lung adenocarcinoma cells. The results indicated that the levels of miR-1246b were low and the levels of FGF14 mRNA were high in H1299 cells and that the expression of miR-1246b was high and the levels of the FGF14 gene were low in H1975 cells. **B**, **C** Cell proliferation was detected by CCK-8 (**B**) and EdU (**C**) assays in H1299 and H1975 cells following miR-1246b inhibitor or mimic treatment. **D** The cell migration ability was evaluated by wound healing assay. **E** Cells were tested for their invasion ability using Transwell assays. **F** qPCR verified the expression of FGF14 mRNA. **G** The expression of FGF14 protein was examined by western blotting. **H** qPCR verified the expression of E-cadherin, N-cadherin, Vimentin, and ERK mRNA in lung cancer cells. **I** The expression of E-cadherin, N-cadherin, Vimentin, p-ERK, and ERK protein was examined by western blotting in lung cancer cells. All the results are representative of three independent experiments. The data are presented as the means ± SDs. *P < 0.05, **P < 0.01 (Student’s *t* test).

To further clarify the regulatory effect of miR-1246b on FGF14 in tumor cells, we detected the expression of miR-1246b and FGF14 in three lung adenocarcinoma cell lines, A549, H1299 and H1975. The expression of miR-1246b was the lowest in H1299 cells and highest in H1975 cells, while the expression of FGF14 was the opposite (Fig. 3A). The subsequent experiments were carried out using H1299 and H1975 cells.

miR-1246b mimics and inhibitors were transfected into H1299 and H1975 cells and cultured for 24 h. The results of the CCK-8 and EdU assays indicated that overexpression of miR-1246b dramatically promoted the proliferation of lung cancer cells and that knockdown of miR-1246b inhibited the proliferation of lung cancer cells (Fig. 3B, C). Wound healing assay results indicated that the overexpression of miR-1246b increased the migration rate of tumor cells. In contrast, the knockdown of miR-1246b reduced tumor cell migration (Fig. 3D). Transwell assays were used to assess the effect of miR-1246b on tumor cell invasion. As shown in Fig. 3, compared with that in the control group, the number of cells in the miR-1246b mimic group that penetrated the lower chamber of the Transwell obviously increased, and the miR-1246b inhibitor group showed the opposite results (Fig. 3E). These results suggest that miR-1246b promotes the proliferation, migration and invasion of lung cancer cells. To clarify the possible mechanism, we further performed western blotting. The data showed that miR-1246b downregulated the expression of FGF14 mRNA and protein in H1299 cells and that the mRNA and protein expression levels of FGF14 increased after the downregulation of miR-1246b expression in H1975 cells (Fig. 3F, G). The overexpression of miR-1246b increased the protein expression levels of p-ERK/ERK, N-cadherin, and Vimentin and decreased the protein expression level of E-cadherin. The qPCR results showed the same tendencies, with the exception of a decrease in the expression of E-cadherin at the mRNA level. Therefore, we deduced that miR-1246b may promote tumor development by ERK and EMT alterations through FGF14 (Fig. 3H, I).

### FGF14 inhibits the proliferation, migration and invasion of lung cancer cells

**Fig. 4:**
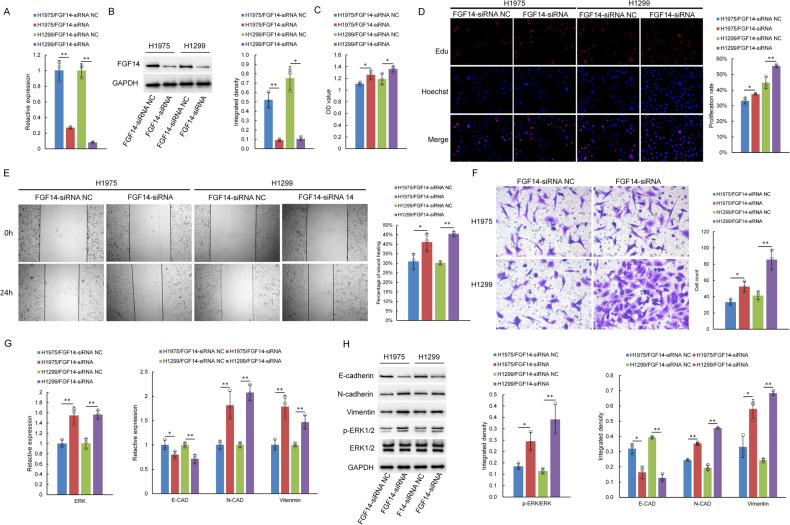
FGF14 downregulation promoted cell proliferation, migration and invasion in H1299 and H1975 cells. The FGF14 gene was silenced in H1299 and H1975 cells. **A** FGF14 mRNA expression examined by qPCR in 2 cell lines. **B** FGF14 protein expression examined by western blotting. **C**, **D** Cell proliferation was assessed by CCK-8 assay (**C**) and EdU assay (**D**). **E** The cell migration ability was examined by wound healing assay. **F** H1299 and H1975 cells were assessed for their migration ability using a Transwell assay. **G** qPCR was performed to determine E-cadherin, N-cadherin, Vimentin, and ERK mRNA levels in H1299 and H1975 cells. **H** The expression of E-cadherin, N-cadherin, Vimentin, p-ERK, and ERK protein was analyzed by western blotting in H1299 and H1975 cells. All the results are representative of three independent experiments. The data are presented as the means ± SDs. *P < 0.05, **P < 0.01 (Student’s *t* test).

Previous studies have found that FGF14 can inhibit the proliferation of lung cancer cells. In this experiment, plasmid transfection was used to downregulate the expression of FGF14 in H1299 and H1975 cells (Fig. 4A, B). The results of CCK-8 and EdU assays showed that after inhibiting the expression of FGF14 in lung cancer cells, the proliferation rate of tumor cells significantly increased (Fig. 4C, D), and the wound healing assay results demonstrated that the migration ability of lung cancer cells was enhanced (Fig. 4E). Transwell experiments also suggested that compared with that in the control group, the number of tumor cells passing through the chamber was significantly increased after FGF14 expression was inhibited (Fig. 4F). Further analyses found that with decreased FGF14 expression in lung cancer cells, the expression of p-ERK/ERK, N-cadherin, and Vimentin increased, but the expression of E-cadherin decreased (Fig. 4G, H). These results verified that FGF14 could promote the proliferation, migration and invasion of lung cancer cells by regulating the ERK pathway and EMT.

### miR-1246b rescue experiment

**Fig. 5:**
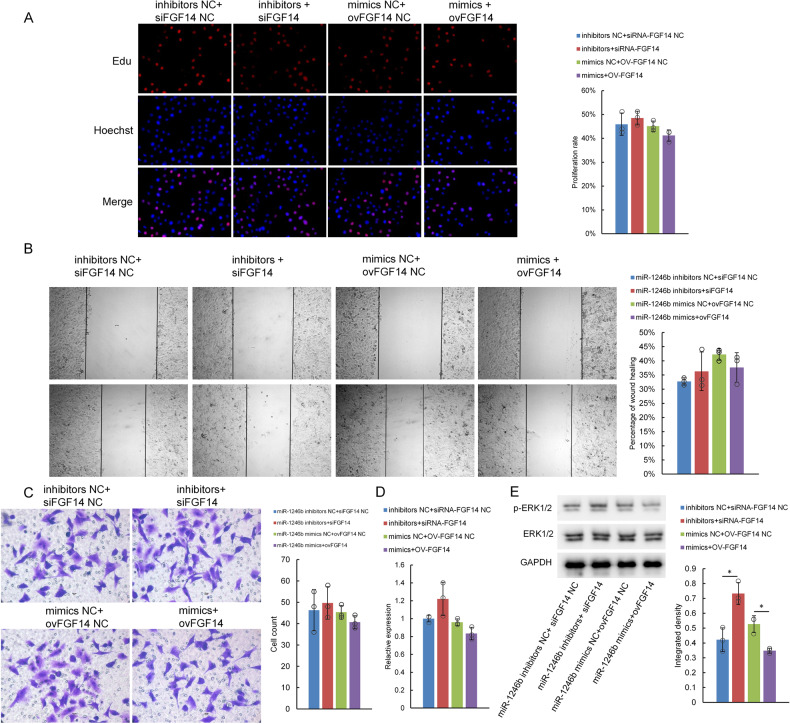
miR-1246b and FGF14 expression rescue could reverse the effect of inhibition of these factors on the malignant behavior in H1299 cells. **A** H1299 cells transfected with miR-1246b inhibitors+siFGF14 or miR-1246b mimics+ovFGF14 were tested for viability using the EdU assay at the indicated time points. **B** A wound healing assay was performed on H1299 cells transfected and treated as indicated. **C** Transwell assays were performed on H1299 cells transfected as indicated. **D** Results of qPCR to measure ERK mRNA levels in H1299 cells treated as indicated. **E** Western blotting was used to measure p-ERK/ERK protein levels in H1299 cells. All the results are representative of three independent experiments. The data are presented as the means ± SDs. *P < 0.05, **P < 0.01 (Student’s *t* test).

The low expression of miR-1246b led to the increased expression of FGF14. We used a si-FGF14 plasmid to rescue the increased expression of the target gene; the overexpression of miR-1246b led to the decreased expression of FGF14, and we employed an FGF14 overexpression plasmid to rescue the reduced expression of target genes. The EdU assay results showed recovery of the cell proliferation rate, and the data of wound healing and Transwell experiments also suggested that the effect of miR-1246b on lung cancer cell migration and invasion abilities was inhibited (Fig. 5A–C). The expression of p-ERK/ERK protein was decreased in FGF14-overexpressing cells; however, in the si-FGF14 group, the above results were reversed. The qPCR analyses showed the same trend (Fig. 5D, E). The above data also suggest that in addition to ERK, there may be other pathways involved in the effect of miR-1246b-FGF14 on the malignant behaviors of lung cancer cells.

### miR-1246b promotes the growth of lung cancer in vivo

**Fig. 6:**
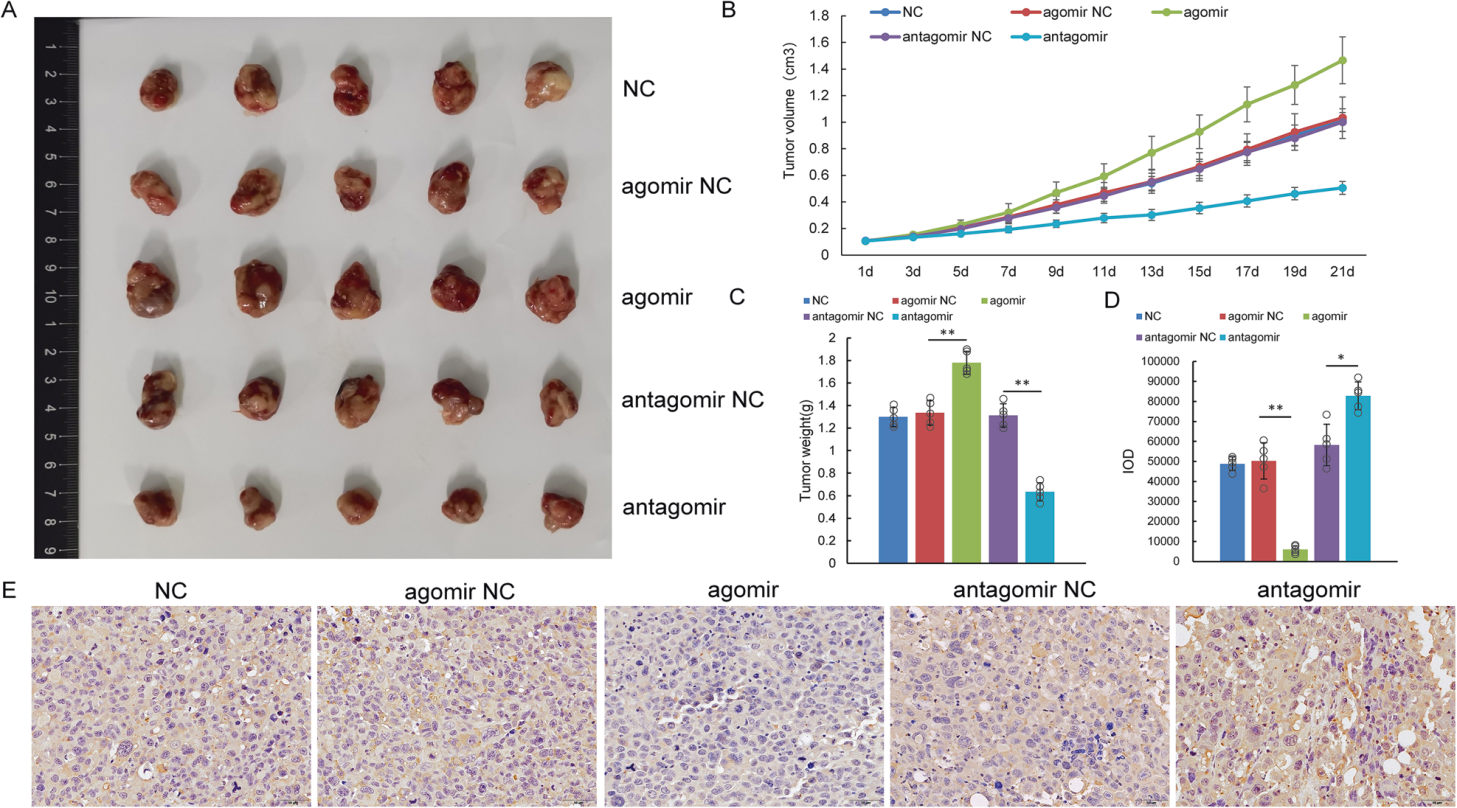
The effect of miR-1246b on H1299 cell NSG mouse xenograft tumors. **A** Samples from mouse xenografts injected with miR-1246b agomir or antagomir. **B**, **C** Tumor volume (**B**) and weight (**C**) in mouse xenografts. **D**, **E** FGF14 expression examined by immunohistochemical staining in tumor tissues. **D** Shows the quantitative analyses based on IOD values obtained from immunohistochemical staining of FGF14 in tumor tissues. All the results are representative of five independent experiments. The data are presented as the means ± SDs. *P < 0.05, **P < 0.01 (Student’s *t* test).

Finally, we established a mouse subcutaneous xenograft model with H1299 cells to verify the underlying mechanisms of miR-1246b-induced tumor growth in vivo. With increasing time, the tumor volume and weight increased significantly in miR-1246b-overexpressing xenograft tumors compared with those of the control group (Fig. 6A–C). Immunohistochemical results indicated that the expression of FGF14 was reduced in tumor tissues with high miR-1246b expression, indicating that miR-1246b could promote tumor proliferation by downregulating the expression of FGF14 (Fig. 6D, E).

## Discussion

MiRNA alterations in EVs have been extensively studied in the diagnosis, treatment and pathogenesis of lung cancers. In addition to the effect on the malignant development of lung cancers, it has also been found that miR-223-3p and miR-29a-3p can inhibit the proliferation and migration of non-small cell lung cancer (NSCLC) cells [27, 28]. The source of these research samples is mainly the peripheral blood of patients. The content of EVs in peripheral blood is higher than that in BALF, but the source is complex, and the heterogeneity is strong [29]; plasma also contains high concentrations of proteins and lipoproteins, making the purification of EVs difficult [30, 31]. However, BALF does not have high lipoprotein and protein contents. Collecting the components in the local bronchoalveolar area, especially the BALF, where the lung nodular lesions are located, can better reflect the local microenvironment [17]. Our research subjects were patients with pulmonary nodules, and accurate positioning is required to obtain BALF of the local lung tissue with nodule lesions. Compared with conventional bronchoscopy, the R-EBUS technology used in our study has more advantages regarding the localization of peripheral lung lesions [32, 33]. Our experiments confirmed that through a standardized protocol, sufficient BALF was obtained, EVs were successfully isolated, and the measured concentration was close to that reported in a previous study [17], demonstrating the feasibility of this method.

The tracer experiment demonstrated that lung cancer cells could take up BALF-EVs from malignant nodules and further increase the proliferation and invasion ability of tumor cells, indicating that BALF-EVs from early-stage lung cancer patients can serve as carriers to deliver the information required for tumor cell proliferation and invasion.

Until now, only one study on miRNAs in BALF-EVs from patients with early-stage lung cancer has been reported. The study suggested that the levels of miR-126 and Let-7a in BALF-EVs from patients with early-stage lung cancer are higher than those in BALF-EVs from controls [25], but this result contradicted blood test data [34, 35]. In vitro experiments have also confirmed that miR-126 and Let-7a inhibit the proliferation, migration and invasion of NSCLC cells [36, 37]. In this study, we sequenced small RNAs in BALF-EVs from patients with benign and malignant nodules. Among the differentially expressed miRNAs, there were previously known miRNAs and novel miRNAs. We verified this finding in samples from 20 pairs of patients, and a new miRNA sequence, miR-1246b, caught our attention. The AUC for miR-1246b in the diagnosis of benign and malignant pulmonary nodules was only 0.743, which may be related to our small sample size. Furthermore, previous studies have found that the diagnostic performance of multiple miRNAs is higher than that of a single miRNA in tumors [38, 39]. Therefore, a follow-up study should be carried out to expand the sample size; a combination with other miRNAs may also be considered.

We initially explored the possible role of miR-1246b in the development of lung cancer. We first proved that miR-1246b could promote the proliferation, migration and invasion of tumor cells by altering the expression of miR-1246b in lung cancer cells. Subsequently, we predicted the possible binding targets of miR-1246b by bioinformatics analyses. FGF14 has been confirmed to inhibit tumor growth in lung adenocarcinoma, and this effect can be achieved by the inhibition of COL11A1 and MUC16 proteins by FGF14 [40]. We also found that the expression of FGF14 protein in malignant pulmonary nodules was lower than that in benign lesions. Further in vitro studies suggested that FGF14 inhibited the proliferation and invasion of tumor cells by inhibiting ERK phosphorylation. To determine whether FGF14 is a target gene of miR-1246b, we performed a dual-luciferase reporter assay, and the results verified that FGF14 can bind to miR-1246b and that the expression of FGF14 increased after inhibiting the expression of miR-1246b. In contrast, the upregulation of miR-1246b could decrease the expression of FGF14 and ultimately enhance the proliferation, migration and invasion of tumor cells by activating ERK and EMT. The upregulation of FGF14 in tumor cells can antagonize the tumor growth-promoting effect of miR-1246b. The results of animal experiments also suggested that miR-1246b decreased FGF14 protein levels and promoted the growth of xenograft tumors, and vice versa. Previous studies have reported that kinases of the ERK family promote EMT and enhance the cell migration and invasion properties of tumor cells after the ERK pathway is activated [41, 42]. Therefore, we speculate that the tumor-promoting process of miR-1246b may be caused by the activation of ERK after binding to FGF14.

**Fig. 7:**
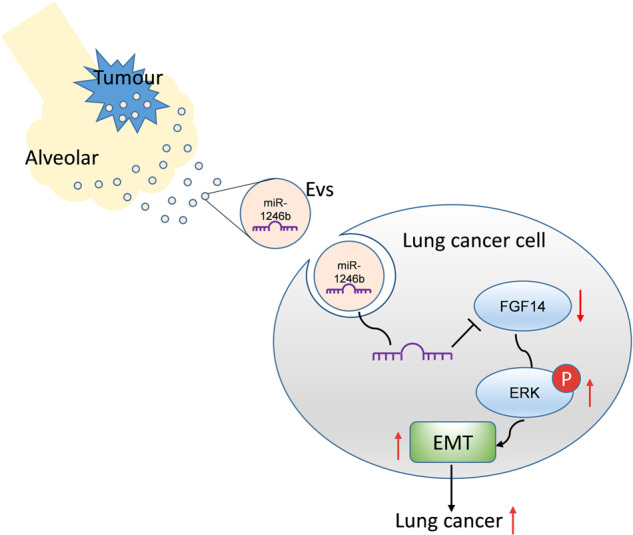
Schematic diagram of the molecular mechanisms by which miR-1246b promotes lung cancer progression. miR-1246b delivered by BALF-EVs promotes lung cancer progression by inhibiting FGF14 and activating ERK.

Notably, our study has some limitations. First, we only studied BALF-EVs and did not use peripheral blood for comparison, and further investigations with larger sample sizes are necessary. Second, because the common pathological type of malignant pulmonary nodules, especially peripheral pulmonary nodules, is adenocarcinoma, there was a predominance of patients with adenocarcinoma included in this study, with only one case of squamous cell carcinoma and one case of small cell carcinoma, and the cell lines selected for the follow-up experiment were also adenocarcinoma cell lines; therefore, the different mechanisms may exist in other types of lung cancer. As mentioned previously, whether other proteins downstream of FGF14 also play a positive role in the ERK pathway needs to be investigated. Finally, BALF-EVs may be derived from a variety of cells; therefore, further studies are needed to isolate EVs from specific cells to identify the source of EVs carrying miRNAs that play an important role in this process (Fig. 7).

In conclusion, our findings suggest that BALF can be used as a source of liquid biopsy for early-stage lung cancer patients and that EVs can be successfully isolated. BALF-EVs carrying miR-1246b activate ERK and EMT by binding to FGF14, ultimately leading to the enhancement of lung cancer cell proliferation, migration and invasion.

### Supplementary information

S Figure Legends

Supplementary Figure 1

Supplementary Figure 2

Supplementary Figur e3

Original drawing

aj-checklist

## Data Availability

The datasets generated and/or analyzed during the current study are not publicly available due ethical reasons but are available from the corresponding author on reasonable request.
