# Supplementary material for: MiR-1246b, a novel miRNA molecule of extracellular vesicles in bronchoalveolar lavage fluid, promotes nodule growth through FGF14 in patients with lung cancer
Source: Cell Death Dis. 2023 Dec 1;14(12):789. doi: 10.1038/s41419-023-06218-9 (PMC10692082; doi:10.1038/s41419-023-06218-9)
Supplement: Supplementary file 1 — S Figure Legends [file 41419_2023_6218_MOESM1_ESM.docx]

**Figure Legends**

Figue1. Analysis clustering heatmaps of known and novel miRNA family

Observing the families of miRNAs in each sample at the sorting level, we select the top 10 families of known and novel miRNAs in each sample, and then take their union for cluster analysis.

Figue2. GO and KEGG enrichment differences for the target genes of known miRNAs.

The top 10 positions were selected for pathway enrichment analysis: GO(A) and KEGG(B); Directed acyclic graph for Cellular component (C), Molecular function (D), and Biological processes (E).

Figue3. GO enrichment differences for the target genes of novel miRNAs.

Directed acyclic graph for Cellular component (A), Molecular function (B), and Biological processes (C).
