# Supplementary figures and images for: MiR-1246b, a novel miRNA molecule of extracellular vesicles in bronchoalveolar lavage fluid, promotes nodule growth through FGF14 in patients with lung cancer
Source: Cell Death Dis. 2023 Dec 1;14(12):789. doi: 10.1038/s41419-023-06218-9 (PMC10692082; doi:10.1038/s41419-023-06218-9)

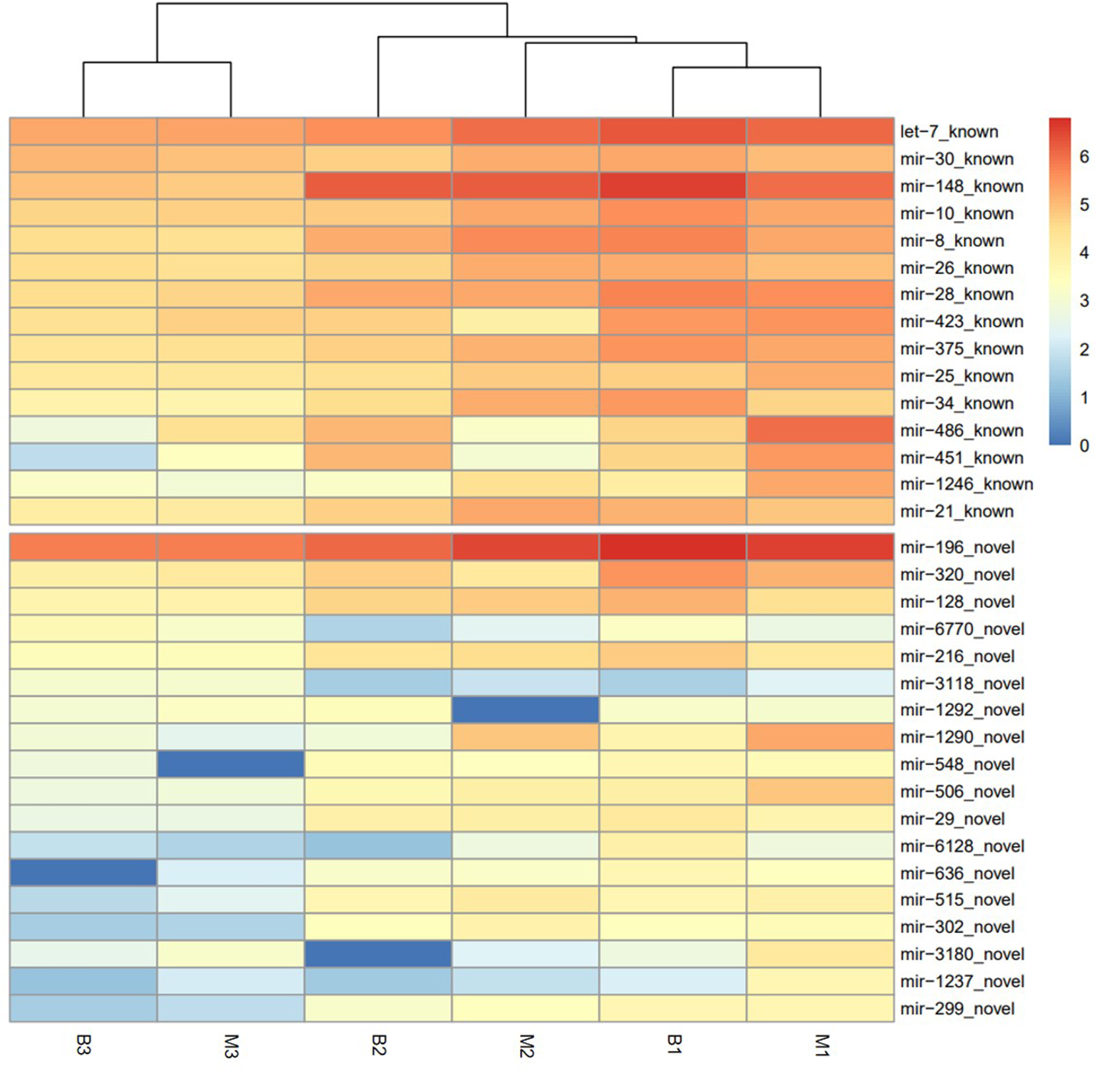

Supplement: Supplementary file 2 — Supplementary Figure 1 [file 41419_2023_6218_MOESM2_ESM.tif]

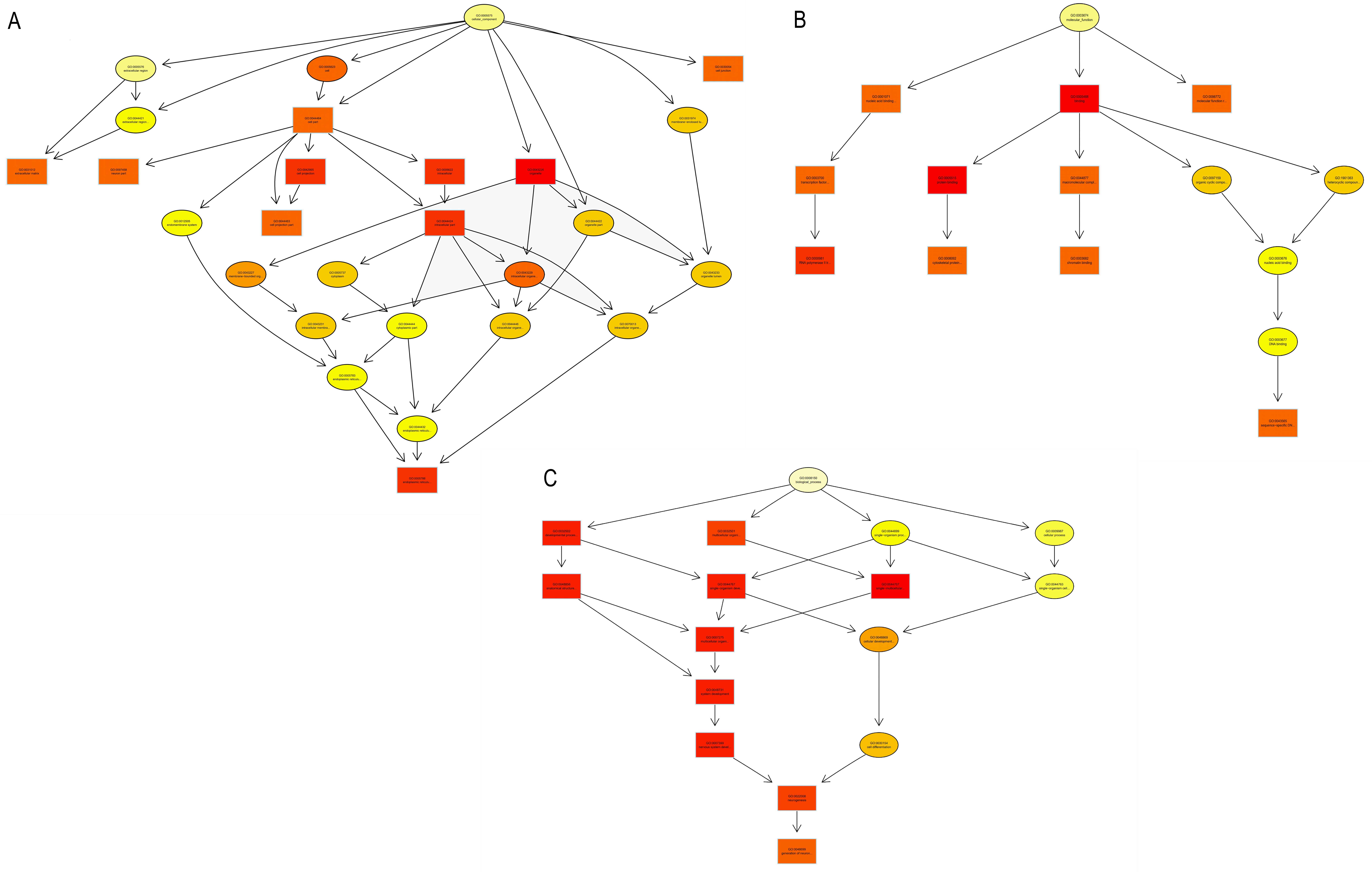

Supplement: Supplementary file 4 — Supplementary Figur e3 [file 41419_2023_6218_MOESM4_ESM.tif]
