# Supplementary material for: MiR-1246b, a novel miRNA molecule of extracellular vesicles in bronchoalveolar lavage fluid, promotes nodule growth through FGF14 in patients with lung cancer
Source: Cell Death Dis. 2023 Dec 1;14(12):789. doi: 10.1038/s41419-023-06218-9 (PMC10692082; doi:10.1038/s41419-023-06218-9)

Senior gel imaging system: Tanon 5200, China

Using ACE FuturePAGE Precast-Gel (F00003Gel, ACE Biotechnology, China)

**Figure 1.** The collection, characterization and the effect of BALF on the proliferatin and invasion in lung cells

**ALIX, Calnexin and CD63**

1-3 samples: Malignant nodules EVs, Benign nodules EVs, HK-2 cell;

4-6 samples: Malignant nodules EVs, Benign nodules EVs, HK-2 cell;

7-9 samples: Malignant nodules EVs, Benign nodules EVs, HK-2 cell.





**ALIX Calnexin**



**CD63**

Senior gel imaging system: SYNGENE G: BOXChemiXR5, UK

Using SDS-PAGE gel preparation kit (KGP113, Key GEN BioTECH, China)

**Figure 3.** miR-1246b may promote cell proliferation, migration and invasion through inhibiting FGF14.

**E-cadherin, N-cadherin, Vimentin, p-ERK, ERK and FGF14**

1-4 samples: H1975/inhibitors-NC, H1975/inhibitors, H1299/inhibitors-NC, H1299/inhibitors;

5-8 samples: H1975/inhibitors-NC, H1975/inhibitors, H1299/inhibitors-NC, H1299/inhibitors;

9-12 samples: H1975/inhibitors-NC, H1975/inhibitors, H1299/inhibitors-NC, H1299/inhibitors.

**
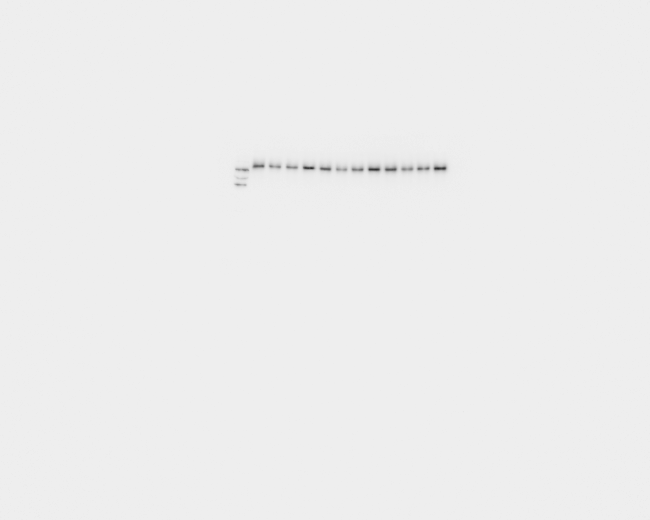
**
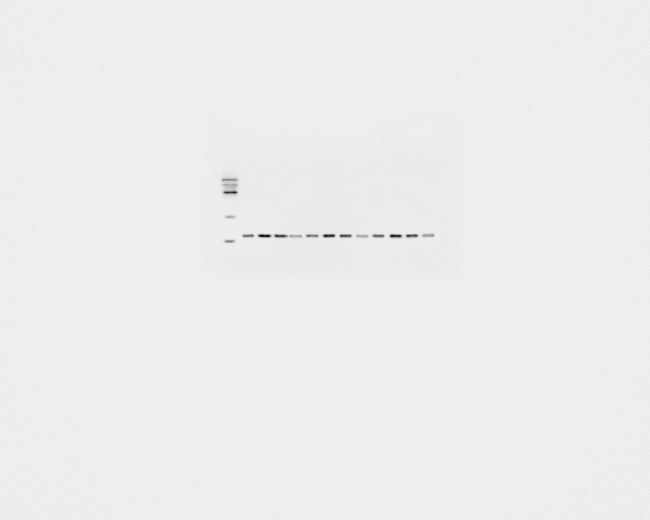
**E-cadherin N-cadherin**


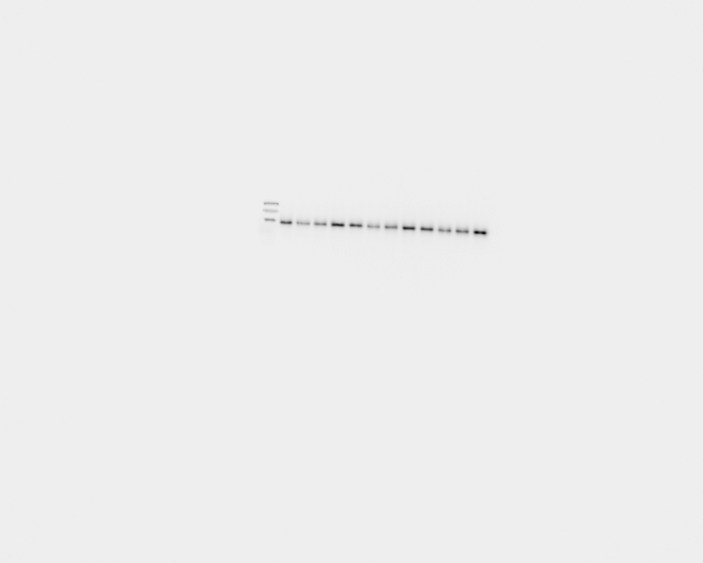


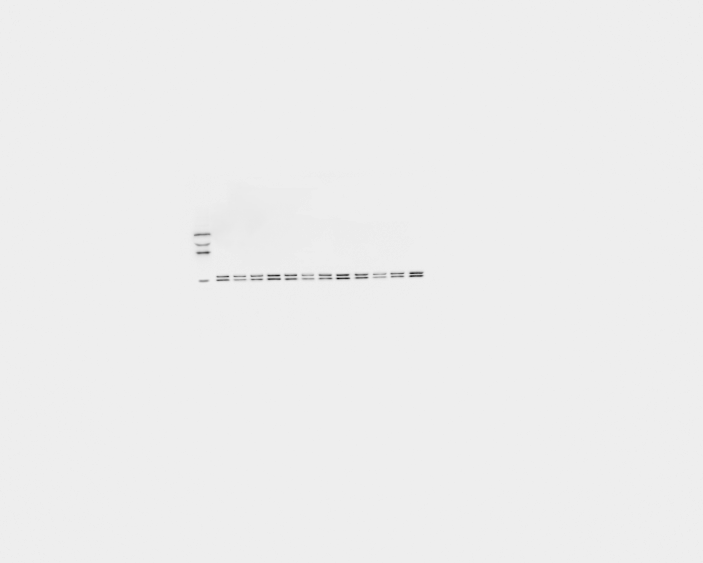
**Vimentin pERK**


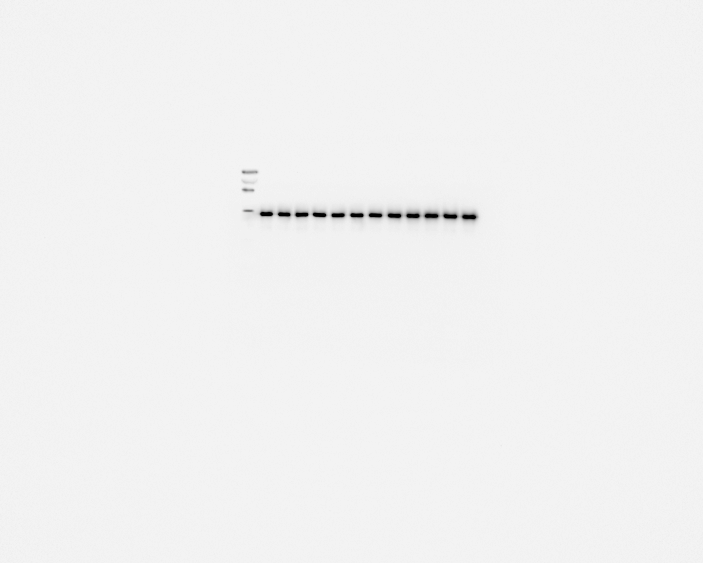
**
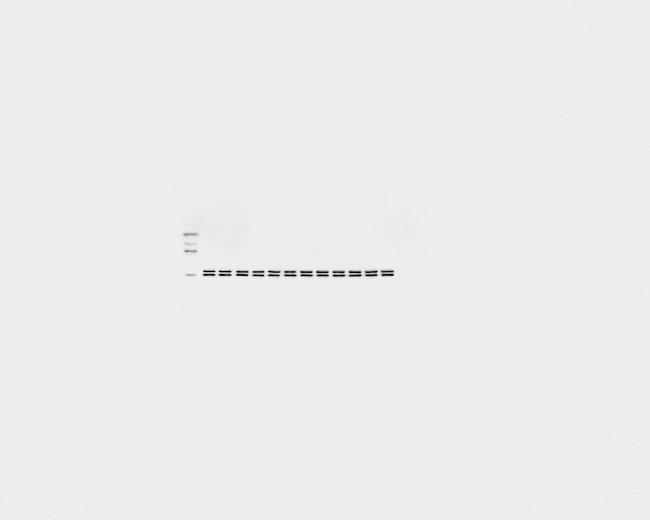
ERK GAPDH**


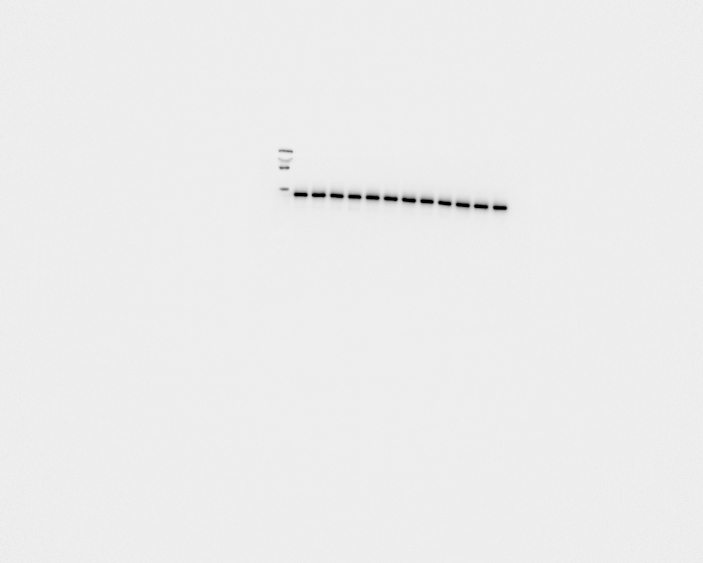
**FGF14 GAPDH**


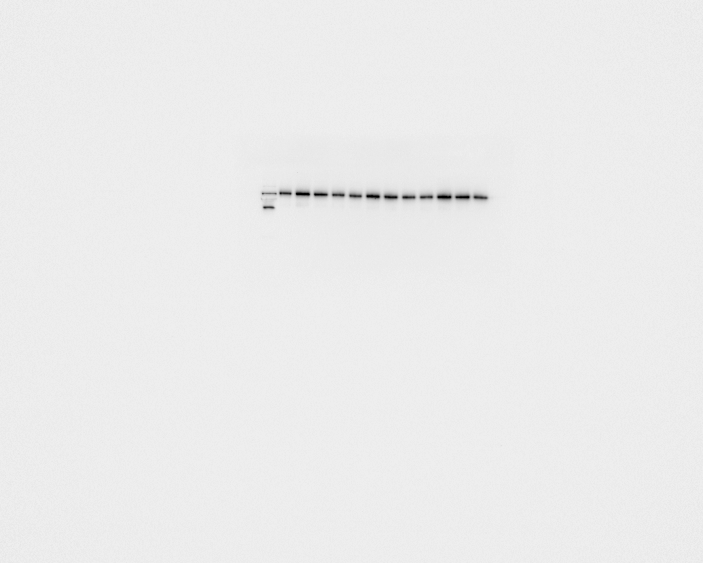


**Figure 4.** FGF14 down-regulation promoted cell proliferation, migration and invasion in H1299 and H1975 cells.

**E-cadherin, N-cadherin, Vimentin, p-ERK, ERK and FGF14**

1-4 samples: H1975/siRNA-NC, H1975/siRNA, H1299/siRNA-NC, H1299/siRNA;

5-8 samples: H1975/siRNA-NC, H1975/siRNA, H1299/siRNA-NC, H1299/siRNA;


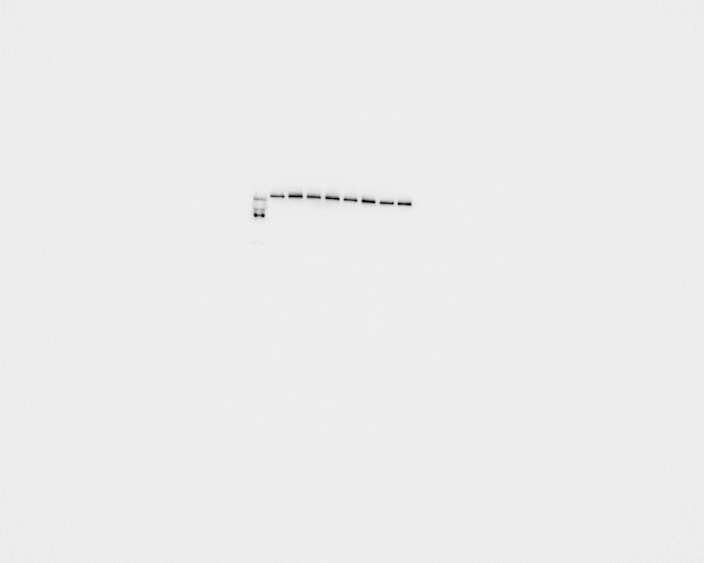

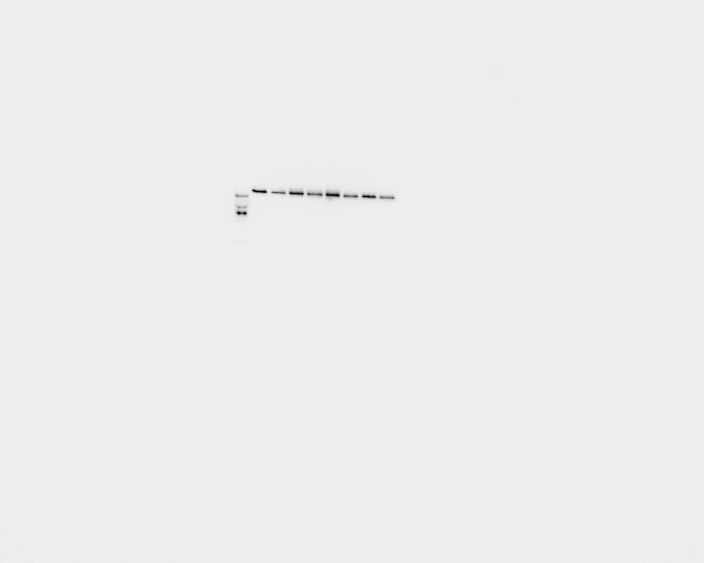
**E-cadherin** **N-cadherin**


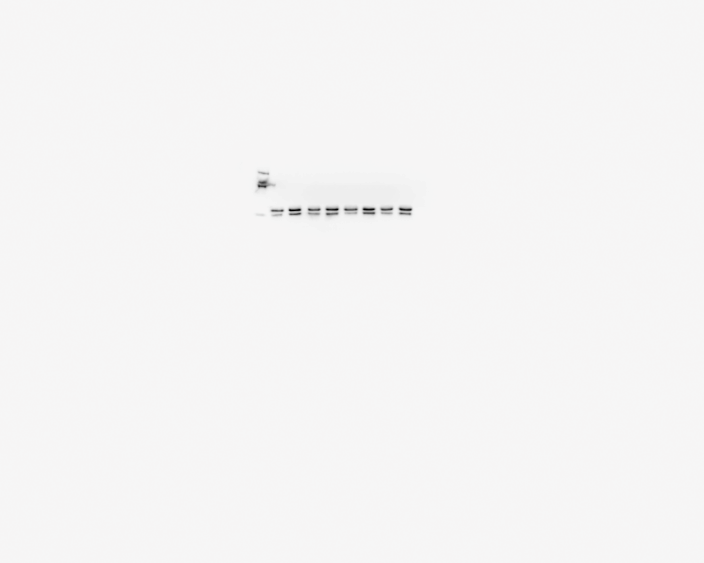

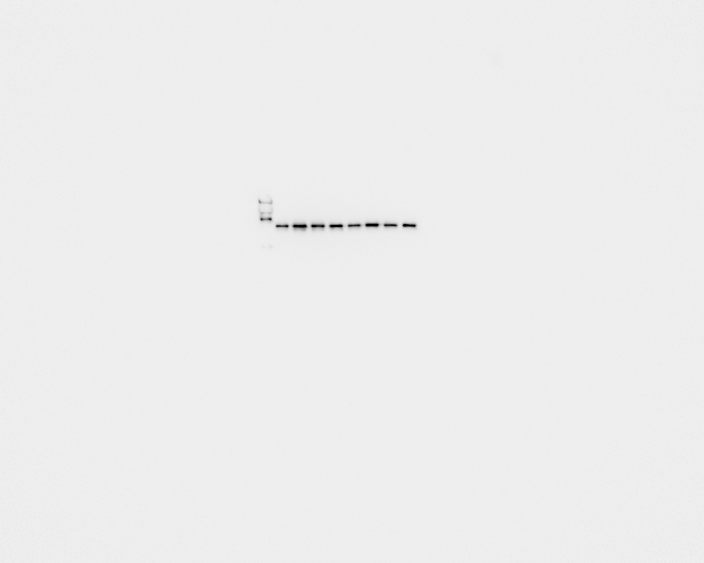
**Vimentin p-ERK**


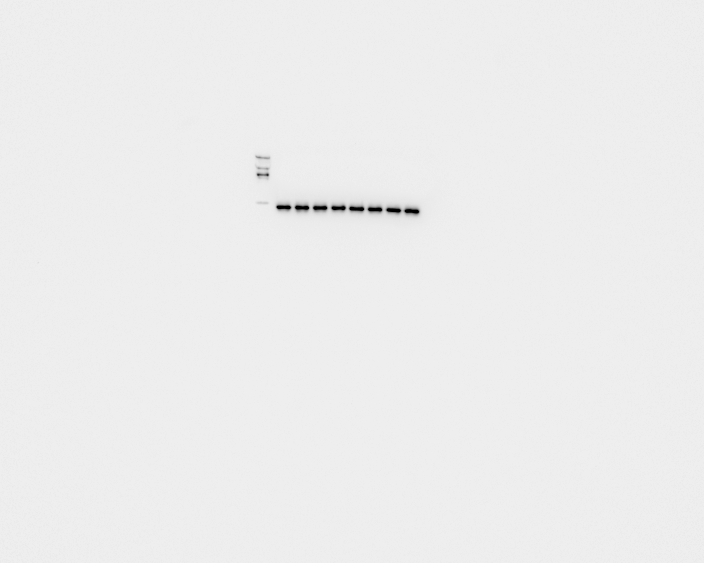

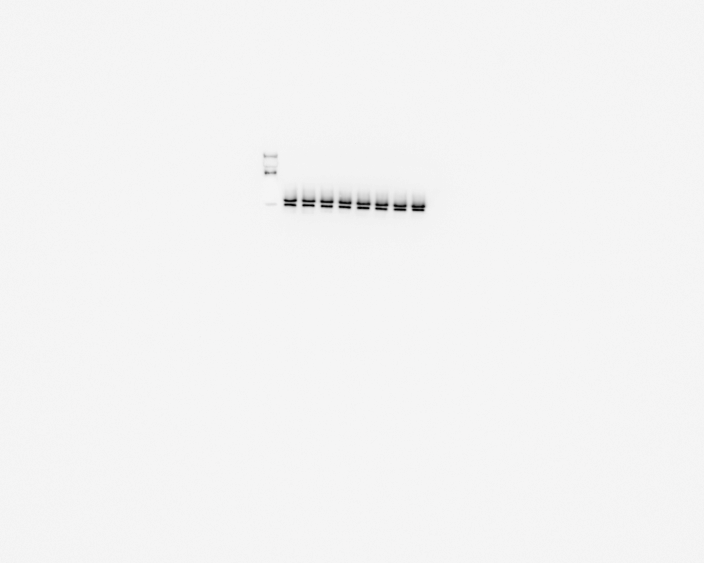
**ERK GAPDH**


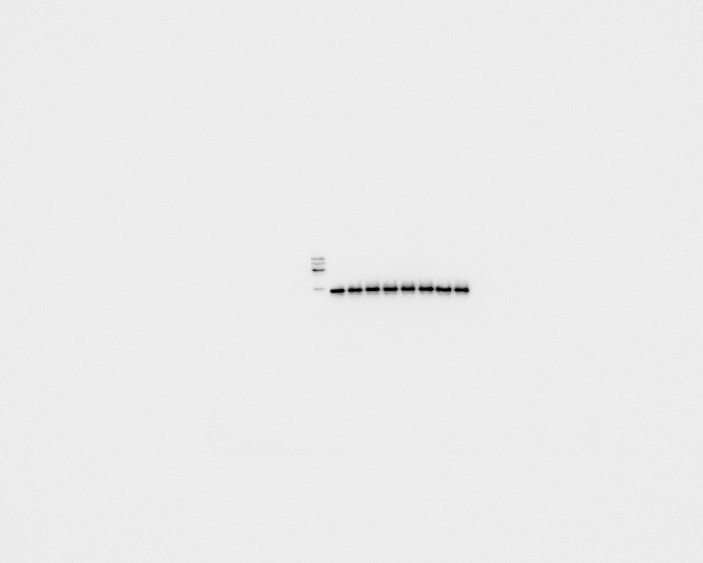

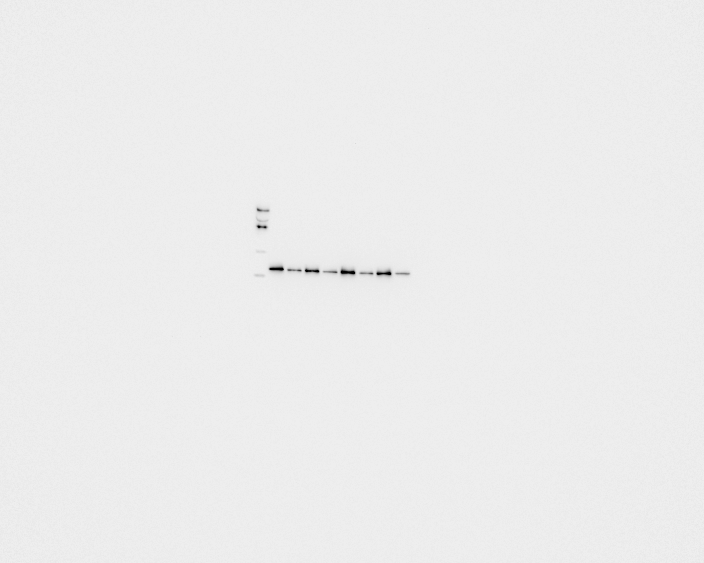
**FGF14 GAPDH**

**Figure 5.** miR-1246b and FGF14 expression rescue could reverse the effect on the malignant behavious in H1299 cells.

**p-ERK, ERK and FGF14**

1-4 samples: inhibitors NC+ siFGF14 NC, inhibitors+ siFGF14, mimics NC+ovFGF14 NC, mimics+ovFGF14;

5-8 samples: inhibitors NC+ siFGF14 NC, inhibitors+ siFGF14, mimics NC+ovFGF14 NC, mimics+ovFGF14;

9-12 samples: inhibitors NC+ siFGF14 NC, inhibitors+ siFGF14, mimics NC+ovFGF14 NC, mimics+ovFGF14.


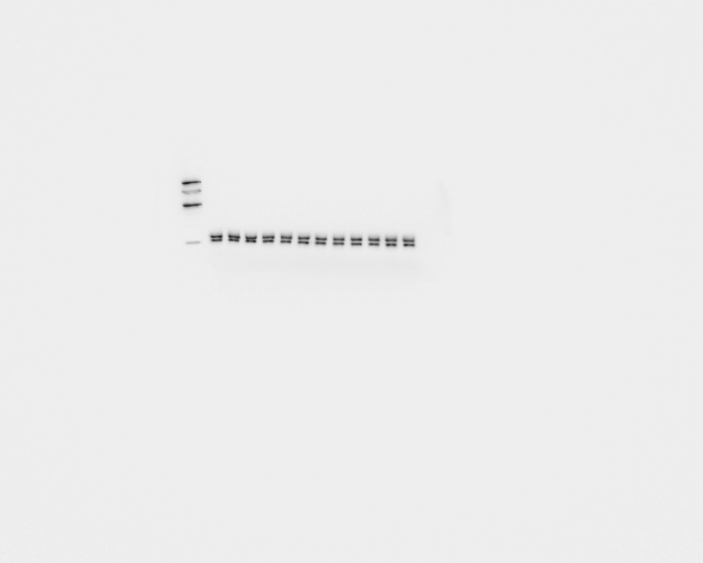
**p-ERK ERK**


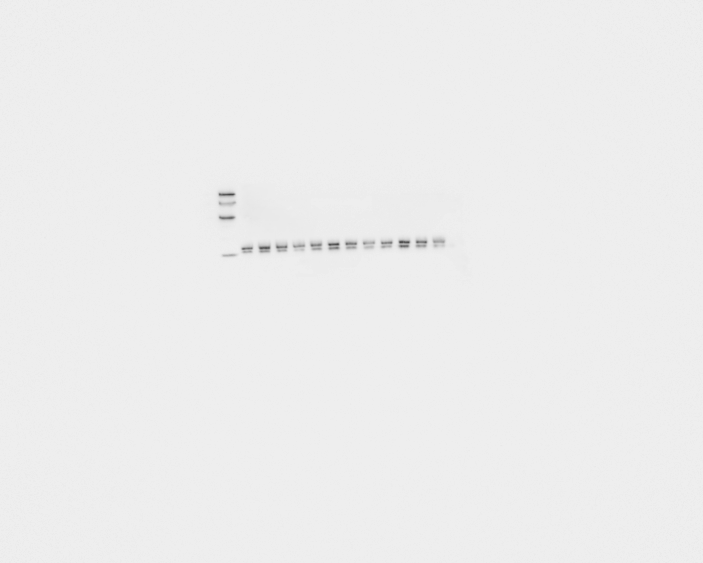


**GAPDH**


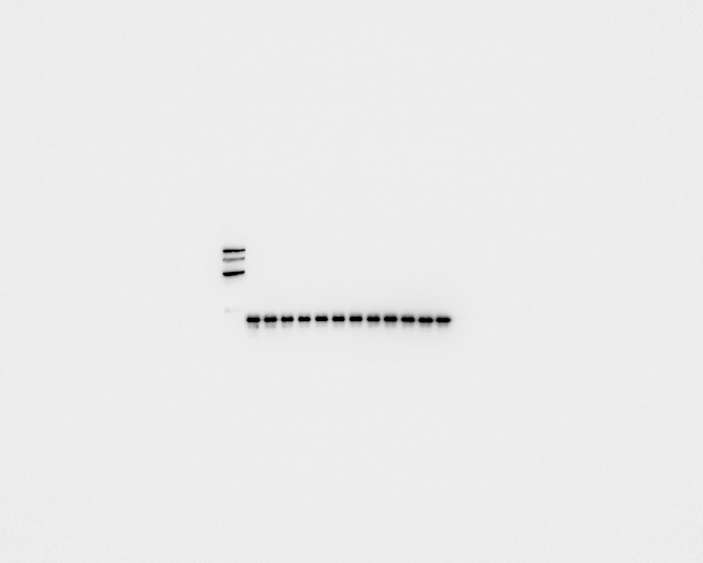

Supplement: Supplementary file 5 — Original drawing [file 41419_2023_6218_MOESM5_ESM.docx]
